# Dynamic role of LMW-hyaluronan fragments and Toll-like receptors 2,4 in progression of bleomycin induced lung parenchymal injury to fibrosis

**DOI:** 10.1186/s43168-021-00073-y

**Published:** 2021-05-21

**Authors:** Apoorva Pandey, Ritu Kulshrestha, Surendra Kumar Bansal

**Affiliations:** 1 Deparment of Pathology, V.P. Chest Institute, University of Delhi, Delhi, 110007 India; 2 Department of Biochemistry, V.P. Chest Institute, University of Delhi, Delhi, 110007 India

**Keywords:** Pulmonary fibrosis, Toll-like receptor 2, 4, LMW-hyaluronan, NF-κB

## Abstract

**Background:**

Pulmonary fibrosis (PF) is a progressive and lethal lung disease of elderly whose incidence has been increasing following the Covid-19 pandemic caused by severe acute respiratory syndrome corona virus 2 (SARS-CoV-2). PF immunopathogenesis involves progressive alveolar epithelial cell damage, release of damage-associated molecular patterns (DAMPs), and extracellular matrix (ECM) injury. We assessed the dynamic role of LMW-hyaluronan (LMW-HA) as DAMP in initiation of host immune TLR-2,4 responses and as determinant in progression of ECM injury to fibrosis. Male Wistar rats were divided into Group I (saline control, *n* = 24) and Group II (intratracheal bleomycin, 7 U/kg/animal, *n* = 24). Animals were euthanized on 0, 7, 14, and 28 days. The time course of release of LMW-HA, TLR-2,4 mRNA and protein levels, and NF-κB-p65 levels after bleomycin injury were correlated with the development of parenchymal inflammation, remodelling, and fibrosis.

**Results:**

Acute lung injury caused by bleomycin significantly increases the pro-inflammatory LMW-HA levels and elevates TLR-2,4 levels on day 7. Subsequently, TLR-2 upregulation, TLR-4 downregulation, and NF-κB signalling follow on days 14 and 28. This results in progressive tissue inflammation, alveolar and interstitial macrophage accumulation, and fibrosis.

**Conclusions:**

LMW-HA significantly increases in PF caused by non-infectious and infectious (Covid-19) etiologies. The accumulating HA fragments function as endogenous DAMPs and trigger inflammatory responses, through differential TLR2 and TLR4 signalling, thus promoting inflammation and macrophage influx. LMW-HA are reflective of the state of ongoing tissue inflammation and may be considered as a natural biosensor for fibrotic lung diseases and as potential therapeutic targets.

## Background

Pulmonary fibrosis is a progressive lung disease characterized by aberrant tissue repair, excessive accumulation of extracellular matrix (ECM), and scarring. It is a recognized sequelae in genetically predisposed individuals undergoing age-related fibroproliferative diseases. It arises from repetitive sub-lethal insults caused by oxidative stress, radiation, chemotherapeutic agents, etc. These varied etiologies show common underlying pathogenesis, alveolar epithelial cell (AEC) injury, epithelial–mesenchymal transition (EMT), and persistent ECM production [1]. Abnormal hyperactive and dysregulated innate immune mechanisms are initiated as a consequence of release of inflammatory cytokines; IL-1β, IL-6, and TNF-α “cytokine storm” and result in (i) acute lung epithelial injury, (ii) release of DAMPs such as low molecular weight-hyaluronan (LMW-HA), heat-shock proteins, high mobility group box protein-1 (HMGB1), etc., (iii) induction of HA synthase 2 (HAS2) in endothelium, lung alveolar epithelial cells, and fibroblasts [2], (iv) dysregulated release of matrix metalloproteinases and ECM remodelling, (v) acute respiratory disease syndrome (ARDS), (vi) epithelial–mesenchymal transition, and (vii) pulmonary fibrosis. The availability of only two antifibrotic drugs till date has highlighted the need to identify the potential clinical and laboratory biomarkers that can predict the subgroup of patients that are going to deteriorate or develop lung fibrosis.

The ongoing Covid-19 pandemic caused by severe acute respiratory syndrome corona virus 2 (SARS-CoV-2) has further increased the occurrence of pulmonary fibrosis since 2020. Diffuse alveolar damage (DAD) caused by SARS-CoV-2 can progress to fibrosis even after virus clearance [3]. Hyaluronan (HA), a highly hygroscopic ECM molecule with the ability to absorb water up to 1000 times its molecular weight, is found in lung alveoli in severe Covid-19 and can promote edema [4]. Since the hyaluronan in cadaveric COVID-19 lung tissue comprises low molecular weight fragments [5], recent studies have suggested estimation of serum and sputum levels of HA at admission to distinguish critically ill patients with Covid-19 infection [5, 6] as well as prove to be a potential therapeutic target [7].

The ECM comprises of fibrous proteins, collagen and elastin, residing in a milieu of glycoproteins, proteoglycans, glycosaminoglycans, growth factors, cytokines/chemokines, proteases, etc [8]. ECM contributes as an active or passive player to diverse cellular processes; differentiation, proliferation, adhesion, migration, and apoptosis [9]. ECM disruption releases hyaluronidases [10], reactive oxygen species [11], and degrades endogenous HA into LMW-HA and HMW-HA fragments [12, 13]. These HA fragments are recognized by cell surface receptors; TLR-2,4, CD44, CD168, layilin, RHAMM [14–18], on the basis of their size and correlate with nature and extent of injury. The LMW-HA vary from a few disaccharides up to over 700 kDa [19] and function as pro-inflammatory DAMPs [20], while the high molecular weight HA (HMW-HA) (> 5000 kDa) signal the resolution of inflammation and injury [14, 21]. Therefore, the type of HA fragments (HMW/LMW) predominating in the tissue after injury act as natural biosensors for the state of tissue integrity [22]. The HA fragments differentially trigger an inflammatory immune response during acute lung infection, and chronic injury/repair [23]. Elevated LMW-HA levels have been reported in sputum of Covid-19 patients [5] and in bronchoalveolar lavage (BAL) fluid of asthma, sarcoidosis, ARDS [24], alveolar proteinosis, IPF patients [25]. BALF elevation of HA is associated with local lung injury while raised levels of HA in blood are indicative of both local lung injury and sequential organ failure. Thus, suggesting the potential utility of HA estimation in identifying local and systemic organ dysfunction in acute respiratory distress syndrome (ARDS) patients [24].

The present study focuses on the pathogenetic pathway of progression of lung tissue inflammation to fibrosis after release of LMW-HA. Elevated LMW-HA release pro-inflammatory cytokines, IL-1β,6, TNF-α [26, 27], and chemokines and facilitates leukocyte access to the injury site.This results in cell proliferation [28], migration [29], dendritic cell activation [17], and sterile inflammation [30]. During stage of chronic inflammation, LMW-HA transcribes matrix metalloproteinases (MMP-1,3,9,10,13), collagen, and cytokines, TGF-β, IL-12, and IGF-I [30–32], resulting in attenuation or progression of ECM remodelling. Further, the LMW-HA fragments act as endogenous ligands for Toll-like receptor (TLR-2 and TLR-4) leading to lung inflammation and injury [14]**.** LMW-HAs engage TLR-2 and activate the macrophage inflammatory response [28]. On the other hand, LMW-HAs engage TLR-4 and protect type-II AECs against oxidant-mediated injury. TLR-4 induction maintains appropriate anti-apoptotic response [33] leading to AEC self-renewal and limiting the extent of fibrosis [34]. The ECM participates in progressive fibrotic scarring of lung by (i) activating a profibrotic feedback loop [35], (ii) abnormal ECM cross-linking resulting in enhanced fibroblast growth and preventing normal ECM turnover in IPF [36]. However, the specific ECM-HA-induced TLR signalling resulting in progression of fibrosis continues to remain an enigma [37].

We propose that the differential host immune response to ECM injury and LMW-HA fragments is the critical determinant of epithelial injury/repair processes outcome after both infectious and non-infectious injurious stimuli. These generate feedback signals, leading to either (i) alveolar macrophage priming, increased TLR-2/4 ratio, basal nuclear factor-kappa B (NF-κB) activation, inflammation, and progression of parenchymal fibrosis, or (ii) reducing oxidative stress, decreased TLR-2/4 ratio, type-II AEC protection, and renewal and repair of lung injury. We elaborate the differential activation of TLRs-2,4 and macrophage influx during bleomycin-induced parenchymal remodelling.

## Methods

### Chemicals

Bleomycin sulfate (Bleocip, Cipla), ketamine hydrochloride, xylocaine, anti-goat-IgG (SAB3700288, Sigma Life Science), TLR-2 (Sc-10739, Santa Cruz, USA), TLR-4 (Sc-16240, Santa Cruz), CD-68 (ab125212, Abcam), NF-κB-p65 (Sc-109, Santa Cruz), ExtrAvidin® Peroxidase (Extra-2,3, Sigma), NovaRED (SK-4800, Vector labs, USA), Meyer’s hematoxylin, TRIzol® (Invitrogen 15596018), chloroform, isopropanol, MMLV (M0253S, NEB), RNase (M0314, NEB), dNTPs (N0447S, NEB), random primers (S1330S, NEB), SYBR Green (S4438, Sigma), protease inhibitor (Sigma), hyaluronan quantikine ELISA (LMW-HA < 35–950 kDa, DHYAL0, R&D Systems, USA), and Lamin-A/C (612162, BD Biosciences, India) were used.

### Animals

Male Wistar rats (150–250 g, *n* = 48) were obtained from the animal house, V.P.Chest Institute. The experimental protocol was approved by institutional animal ethical committee and written consent for use of animals was obtained from IAEC. The animals were divided into two groups, group I: saline control, group II: bleomycin. Both the groups contained 6 animals on each day 0, 7, 14, and 28. Animals were provided with standard rodent diet and water ad libitum. Animal care was as per guidelines laid down by Indian National Science Academy, New Delhi. The experiments were performed in the Animal house of the V.P. Chest Institute. No randomization method and strategy control potential confounders were used.

#### Induction of lung fibrosis

Animals were anesthetized with ketamine hydrochloride (50 mg/kg-b.w, I.M) and local anesthesia with 1% lignocaine. The skin was incised under aseptic precautions and trachea was exposed. In control animals, 100 μl of 0.9% normal saline was instilled intratracheally. Experimental animals received single intratracheal instillation of bleomycin (7 units/kg-bw) in 100 μl saline, as previously described [38]. After instillation, incision was sutured and betadine and antibiotic ointment was applied. Animals were euthanized 0, 7, 14, and 28 days after intratracheal bleomycin administration, by using overdose of ketamine hydrochloride. The lungs were ligated at the trachea and removed en bloc. The lungs were immersed in 10% neutral buffered formalin for fixation and processed through a graded series of alcohols and xylene prior to paraffin embedding. Five-micrometer sections of the lungs were deparaffinized and stained with hematoxylin and eosin stain. The time course of release of LMW-HA fragments, TLR-2,4 mRNA and protein, NF-κB-p65, macrophage influx, and CD68 expression after bleomycin injury were correlated with development of parenchymal inflammation and fibrosis.There were no exclusions in analysis of control and experimental groups.

### LMW-HA

LMW-hyaluronan levels (< 35–950 kDa) were quantitated by using the quantitative sandwich enzyme immunoassay technique (Hyaluronan Quantikine ELISA Kit DHYAL0, R&D systems). Lung tissue (500 mg) was homogenized in lysis buffer (0.5% TritonX-100, 150 mMNaCl, 15 mM Tris, 1 mM CaCl_2_, 1 mM MgCl_2,_ pH 7.40) and centrifuged at 12,000*g* (4 °C, 20 min). Fifty-milliliter aliquots of supernatant sample were pipetted into the pre-coated wells. After binding and washing steps, 100 μL of enzyme-linked polyclonal antibodies specific for LMW-hyaluronan was added to the wells. The plates were incubated for 2 h at 37 °C. The unbound antibody-enzyme reagent was removed by washing and a chromogen substrate solution was added. The plates were incubated at room temperature for 30 min. The reaction was terminated with 100 μl of diluted hydrochloric acid solution per well and read at 450 nm in an ELISA reader.

### Gene expression

Total RNA was extracted from lung using guanidinium thiocyanate-phenol-chloroform extractionand reverse-transcribed to cDNA. cDNA was amplified: PCR activation (95 °C, 5 min); 35 cycles of denaturation @ 95 °C (30 s), annealing @ 60 °C (35 s), extension @ 72 °C (30 s); final extension @ 72 °C (7 min). Quantitative real-time PCR was performed using Mastercycler, Eppendorf, and primers: TLR-2: Forward-Primer-ATGGCAGCTCCAGGTCTTTC, Reverse-Primer-TTCCGCTGGACTCCAATGTC, TLR-4: Forward-Primer-TCAAGCCCAAGCCTTTCAGG, Reverse-Primer-TTCTCCCAAGATCAACCGATGG, β-actin: Forward-Primer-GACCTTCAACACCCCAGCCA, Reverse-Primer-GTCACGCACGATTTCCCTCTC. Relative gene expression was calculated, using ΔΔCt method.

### TLR protein

Immunohistochemistry was performed on lung sections which were deparaffinized and rehydrated through graded alcohols. Endogenous peroxidase was quenched by treatment with 0.3% hydrogen peroxide in methanol for 3 min. Sections were incubated with the primary antibodies—TLR-2, TLR-4, CD68. The bound antigen was then visualized with the avidin-biotinylated peroxidase technique using DAB substrate. Sections were counterstained with Harris’ hematoxylin, dehydrated, cleared in xylene, and mounted with DPX. Immunostaining was quantified using a Nikon-90i microscope and NIS-Ar image analysis software as per previously described method [39]. Briefly, 10 fields (× 40) were randomly selected and chromogen-positive cells measured. The intensity of positively stained cells was subtracted from 250 (maximum intensity of RGB image) to obtain reciprocal intensity which is directly proportional to protein expression.

### NF-κB-p65

NF-κB-p65 was assessed in lung tissue nuclear extracts by Western blot as per previously described method [40]. Then, 200 mg tissue was homogenized in buffer-A (150 mM NaCl, 0.5 mM PMSF, 1 mM EDTA, 10 mM HEPES, 0.6% NP-40). The nuclear pellet was resuspended in solution-B (25% glycerol, 20 mM HEPES, 420 mM NaCl, 1.2 mM MgCl_2,_ 0.2 mM EDTA, 0.5 mM PMSF, 0.5 mM DTT). Total nuclear proteins were quantified using Bradford assay [41]. Proteins were resolved on 12% SDS-PAGE and transferred onto PVDF membranes. Membranes were blocked with 5% skimmed milk in TBST buffer and incubated with 10 μl of primary antibodies (1:1000), NF-κB-p65, and laminin-A/C (1:1000) for 2 h at room temperature. Membrane was washed thrice with TBST and then incubated with biotinylated secondary antibody (1:2000) of goat anti-Rabbit IgG for 2 h. Following washing, membrane was incubated with extravidin (1:5000) for 2 h and visualized using NovaRED in Gel documentation system (Bio-Rad). Membrane was then blocked again with 5% skimmed milk in TBST at 4 °C overnight and re-probed with housekeeping protein (Lamin-A/C, 612162, BD Biosciences, India). Densitometry was performed using Image lab software-2.0, and values were normalized to Lamin-A/C.

### Statistics

Statistical analysis was done by GraphPad prism-5.0, using one-way ANOVA with Newman Keule’s post hoc test and expressed as Mean ± SEM (standard error of mean). *P* value < 0.05 was considered significant.

## Results

### Bleomycin-induced LMW-HA

Bleomycin-induced lung injury is characterized by HA fragmentation that act as endogenous ligands for TLRs [17]. Other endogenous ligands of TLRs include fibrinogen [42], surfactant protein-A [43], extradomain-A of fibronectin [44], heparan sulfate [45], and HMGB-1 [46]. These ligands induce innate and adaptive immune response through induction of costimulatory molecules in antigen-presenting cells [47] and propagate parenchymal inflammation [48

**Fig. 1:**
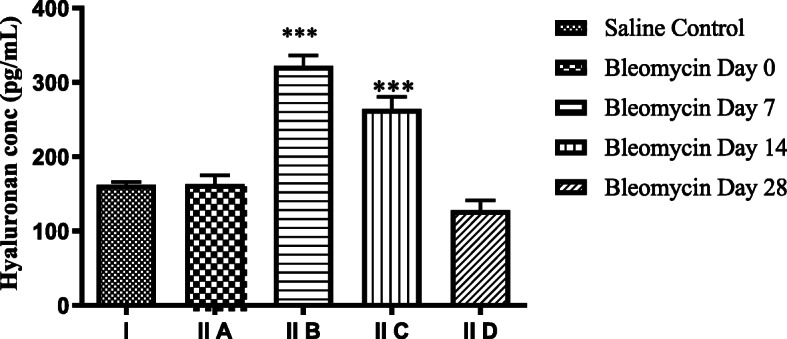
Hyaluronan fragments in lung tissue before and after bleomycin instillation. Significant increase in levels of HA fragments levels on day 7 that persist on day 14 and reduce to baseline levels on day 28. ^***^p < 0.0001 group II B and group II C vs. group II A and group I

The present study shows significant increase in levels of LMW-HA fragments in lung tissue on day 7 (322 ± 14.0 pg/mL), after bleomycin as compared to control (162.2 ± 3.79 pg/mL) (Fig. 1). LMW-HA increased persistently up to day 14 (264 ± 16.65 pg/mL) and decreased in fibrotic phase (day 28, 128 ± 13.15 pg/mL). This is similar to previous study by Teder et al. in mouse model, who observed massive accumulation of HA (5.4 × 10^5^ MW), on day 7, in alveolar spaces and interstitium, following bleomycin, as compared to control (14.4 × 10^5^ MW). They reported that a vast majority of HA fragments are cleared from the lung within 14 days after injury and impaired clearance is followed by collagen deposition and fibrosis [49]. LMW-HA clearance occurs after their internalization [50] by receptors such as TLR2, TLR4, and CD4 [51, 52]. Persisting HA has pro-inflammatory effects and perpetuates tissue inflammation and injury [53]. In a recent autopsy study, hyaluronan staining confirmed prominent HA exudates in alveolar spaces of Covid-19 lungs, suggesting its role in ARDS caused by SARS-CoV-2 [7].

### Bleomycin-induced lung inflammation

**Fig. 2:**
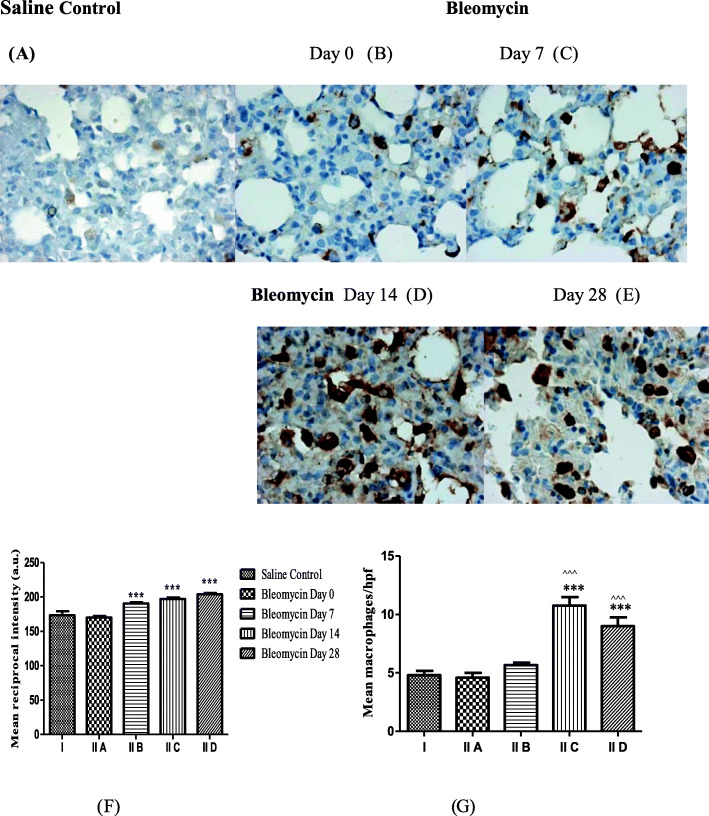
**a** Macrophage infiltration in lung parenchyma before and after bleomycin instillation. Weak CD68 positive macrophages were seen in saline control lungs and on day 0 bleomycin (**a**, **b**) by immunohistochemistry. **c** Note the increase in CD68 expression and influx of macrophages on day 7 after bleomycin instillation (**c**) that persists up to days 14 and 28 (**d**, **e**). **f** Quantification of CD68 protein expression by macrophages shows significant increase in CD68 protein intensity from day 7 onwards up to day 28. ^***^p < 0.0001 group II B, group II C, group II D vs. group II A, group I. **g** A significant net influx of macrophages/high power field (× 400) is seen after bleomycin instillation by morphometry on day 14 that persisted up to day 28. ^***^p < 0.0001 group II C, group II D vs. group II A, group I; ^^^^^*p* < 0.0001 group II C, group II D vs. group II B

Bleomycin causes oxidative damage to AECs DNA, leading to an initial neutrophil influx, followed by infiltration of lymphocytes and macrophages from day 3 onwards [54]. In the present study, we demonstrated chronic interstitial inflammation comprising of lymphocytes and macrophages on days 7 and 14 after bleomycin (Fig. 2d, e) as compared to control (Fig. 2a, b). This was associated with increase in LMW-HA (Fig. 1). This is similar to previous studies, where LMW-HA expression coincides with recruitment of circulating monocytes [55] and early macrophage accumulation at site of lung injury [56]. These CD68-positive macrophages localize to perivascular sites of injury on day 7 after bleomycin [57] and undergo proliferation, M1/M2 polarization, and release profibrotic cytokines like TGF-β1. TGF-β1 activates fibroblasts, causing EMT and ascending grade of parenchymal fibrosis [58]. In the present study, the parenchymal remodelling on day 28 was characterized by reduced cellularity with persistence of macrophages (Fig. 2f) even after LMW-HA levels declined (Fig. 1). LMW-HA and TLR-2,4-induced macrophage macrophage influx and accumulation [14] is suggested to be key component in progression of lung fibrosis [59]. However, LMW-HAs can also stimulate macrophages independently of CD44 and TLR-4 via the TLR-2/MyD88 pathway [19] leading to IRAK, TRAF6, and NF-κB activation [28]. These accumulating macrophages and their associated hyperactive and dysregulated innate immune response need to be explored as biomarkers of disease activity and progression [38]. The innate and adaptive immune imbalance results in unbridled production of pro-inflammatory cytokines and chemokines and contributes to “cytokine storm” and severity of Covid-19 patients [60, 61].

### Bleomycin-induced TLR-2 response

During inflammation, HA fragments differentially engage TLRs, based on their size. HA fragments bind to TLR-2 on alveolar macrophages, trigger NF-κB activation, provide a supportive environment for the immune cells, and promote inflammation [62]. On the one hand, the TLR-NF-κB pathway is central in promoting infection-induced lung injury while on the other hand, increased uptake of HA by macrophages can help in reducing inflammation and promoting repair; therefore, the exact role of TLRs, as a friend or foe in pathogenesis of lung fibrosis, remains to be elaborated [63].

**Fig. 3:**
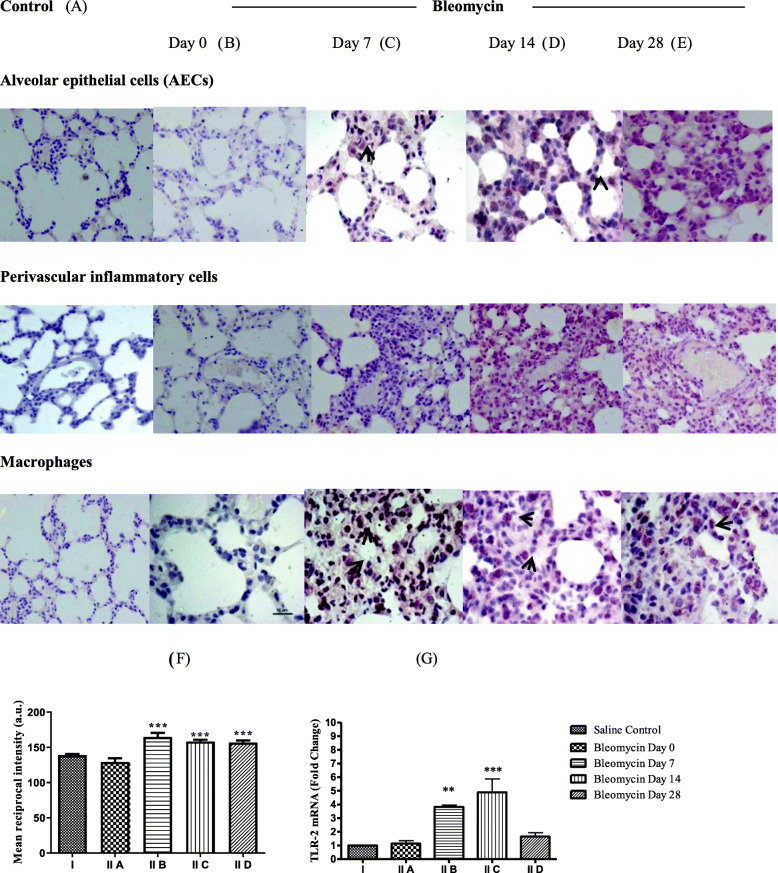
Toll-like receptor-2 (TLR-2) mRNA and protein expression in lungs before and after bleomycin instillation: As compared to saline control and bleomycin day 0 (**a**, **b**), on day 7 and 14 after bleomycin instillation, an increased TLR-2 expression is seen in AECs, perivascular inflammatory cells, alveolar and interstitial macrophage by immunohistochemistry (**c**, **d** respectively). **e** On day 28, in fibrotic phase, TLR-2 protein expression persisted in AECs, alveolar and interstitial macrophages of lung parenchyma. **f** Quantification of the intensity of TLR-2 protein expression in the lung parenchyma. Significant increase in TLR-2 protein expression was seen from day 7 that persisted up to day 28. ^***^p < 0.0001 group II B, group II C, group II D vs. group II A and group I. **g** TLR-2 mRNA levels were significantly upregulated on day 7 and day 14 and returned to baseline on day 28. ^**^p < 0.001 group II B vs. group II A; ^***^p < 0.0001 group II C vs. group II A

In present study, significant increase in TLR-2 mRNA (Fig. 3g, fold change (FC)-3.8, ^**^p < 0.001) and protein expression (Fig. 3c) was seen in AECs, perivascular inflammatory cells, and macrophages, on day 7, after bleomycin, as compared to control (Fig. 3a). On day 14, TLR-2 mRNA levels remained elevated (Fig. 3g, FC-4.8, ^***^p < 0.0001) and correlated with its enhanced protein expression in all above cell types (Fig. 3d). The significantly increased TLR-2 mRNA levels on days 7 and 14 correlated with elevated LMW-HA levels on these days (Fig. 1). Upregulated TLR-2 mediates production of TGF-β1 and interleukins, IL-6,12,23 [9, 64], and initiates the Th2-lymphocyte response [65]. From the resulting chemokine production, M2 macrophage polarization leads to cellular phase of bleomycin-induced pneumonitis [66]. On day 28, TLR-2 mRNA levels decreased as compared to control (Fig. 3g, FC-1.65); however, TLR-2 protein expression persisted in AECs and macrophages (Fig. 3e,f) and was associated with persistent M2 macrophage polarization and progression of tissue fibrosis [67]. HA-TLR2 binding activates NF-κB, MAPKs, p38, and JNK pathways and releases pro-inflammatory and profibrotic cytokines such as interleukin-1, MIP-1, PDGF, and TGF-β1 [68]. Previously, our group has demonstrated an increased expression of TGF-β1 in type-II AECs, EMT cells, alveolar macrophages, and interstitial fibroblasts from day 7 up to day 35 after bleomycin [69]. Thus, LMW-HA-TLR-2 interactions are not only critical as pro-inflammatory signalling cascade but are also associated with increased TGF-β1 expression [69]. Blocking this pathway may attenuate lung inflammation and fibrosis by altering the pulmonary immune microenvironment [70].

### Bleomycin-induced TLR-4 response

LMW-HA are mainly TLR-4 dependent [15] and upregulate CD68 expression in macrophages in a TLR-4-dependent manner similar to bacterial lipopolysaccharide [71] and interferon-*γ* [72]. The activated macrophages use HA as a substrate to aid in migration towards site of injury, and HA binding helps in retaining the activated cells at the sites of inflammation. The SARS-CoV-2 spike protein strongly interacts with the Toll-like receptor 4 (TLR4) pathway producing pro-inflammatory cytokines such as interleukin-6 (IL-6) and tumor necrosis factor-alpha (TNF-α) culminating in cytokine storm and multiple organ failure [73]. TLR-4 deficiency increases the inflammatory response elicited by LMW-HA [74] resulting in elevated cytokine and chemokine levels [71], which skew towards a Th2/Th1 response and increased fibrosis.

**Fig. 4:**
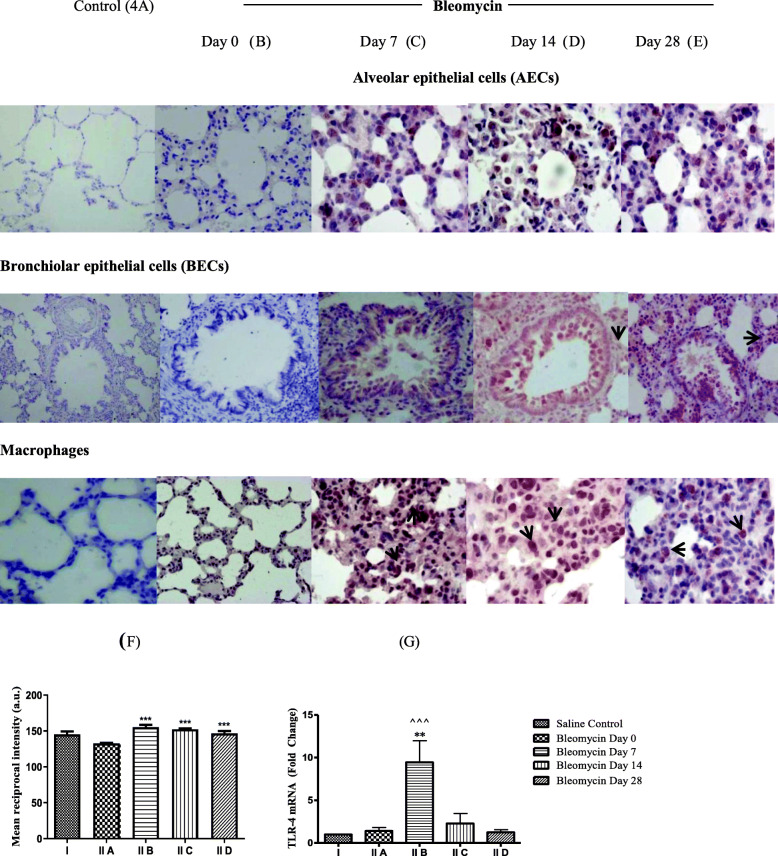
Toll like receptor-4 (TLR-4) mRNA and protein expression in lungs before and after bleomycin instillation. As compared to saline control and bleomycin day 0 (**a**, **b**), on day 7 and 14 after bleomycin instillation, an increased TLR-4 expression is seen in AECs, bronchiolar epithelial cells, and alveolar and interstitial macrophages, on day 7 and on day 14 after bleomycin instillation (**c**, **d** respectively). **e** On day 28, TLR-4 protein expression persists in AECs, bronchiolar epithelial cells, and alveolar and interstitial macrophages. **f** Quantification of the intensity of TLR-4 protein expression in the lung parenchyma. Significant increase in TLR-4 expression was seen from day 7 onwards that persisted up to day 28. ^***^p < 0.0001 group II B, group II C and group II D vs. group II A and group I. **g** TLR-4 mRNA levels were upregulated on day 7 and returned to baseline on day 14 and day 28. ^**^p < 0.001 group II B vs. group II A; ^^^^^*p* < 0.0001 group II B vs. group II D and group I

In the present study, increased TLR-4 mRNA (Fig. 4g, FC-9.4, ^**^p < 0.001) and protein expression was seen in AECs, BECs, and macrophages (Fig. 4c) on day 7, after bleomycin as compared to control (Fig. 4a, f). On day 14, TLR-4 mRNA levels decreased (Fig. 4g, FC-2.29, *p* = ns), while TLR-4 protein expression persisted in AECs, BECs, and macrophages (Fig. 4d) up to day 28 (Fig. 4e). TLR-4 mRNA downregulation correlated with the progression of fibrosis (Fig. 4g, e). TLR-4 protects against oxidant-mediated lung injury by maintaining anti-apoptotic responses [75], promoting alveolar stem cell renewal [33] and epithelial self-defense mechanisms through TLR-4-dependent basal activation of NF-κB [34]. Studies in bleomycin challenged TLR-4 knockout mice have found them to develop stronger inflammatory response [71] with significantly lower type-I collagen mRNA levels as compared to WT mice [76]. The basal TLR-4 activity is critical for resolution of acute and chronic inflammation in pulmonary fibrosis [77]. Our group has previously demonstrated reduction of caveolin-1 levels in bleomycin-instilled lungs [78]. Thus, the TLR-4 mRNA downregulation and accompanying caveolin deficiency [78] contribute to progression to fibrosis during lung injury [79].

### Bleomycin induced NF-κB signalling

**Fig. 5:**
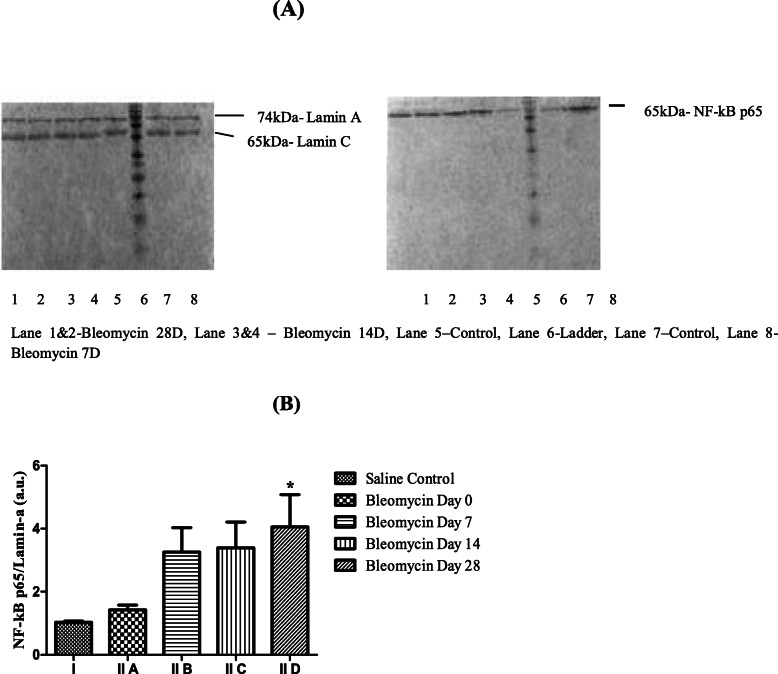
**a** NF-κB p65/Lamin-A/C expression before and after bleomycin treatment. An upregulation of NF-κB p65 expression is seen on day 7 after bleomycin instillation that persists up to day 14 and further increases on day 28, compared to control. **b** Densitometric analysis of the NF-κB p65 and Lamin-A/C (74 kDa and 65 kDa respectively) protein bands shows significant upregulation of NF-κB p65 protein expression from day 7 onwards up to day 28. ^**^p < 0.001 group II C, D vs. group I; ^*^p < 0.05 group II B vs group I

NF-κB activation is induced by HA fragments [80] and TLR-2,4 activation and results in downstream stimulation of TNF-α, TGF-β, and IFN-γ [81]. In the present study, a significant increase of NF-κB-p65 levels were observed from day 7 onwards up to day 28 after bleomycin as compared to control (Fig. 5a, b). This is similar to a previous study which found maximal nuclear translocation of NF-κB-p65 on day 7 after bleomycin instillation [82]. NF-κB-p65 upregulation correlated with perivascular lymphocytes and interstitial macrophage infiltration, in the cellular phase. These alveolar macrophages function as the “first responders,” resulting in the production of cytokines that then activate NF-κB in other cell types [83]. After nuclear translocation, the NF-κB transcriptionally regulates (i) TGF-β1 resulting in fibroblast proliferation [84], (ii) matrix metalloproteinases (MMPs) [85] and their inhibitors, tissue Inhibitor of Matrix Metalloproteinases(TIMPs), resulting in protease-antiprotease imbalance, ECM deposition, and matrix remodelling. In the previous study by our group, we have demonstrated that it is the shift in the balance of MMP-9/TIMP-1,3 ratio to less than 1 that primes the inflammatory response and its progression to fibrosis [86]. Thereby suggesting that NF-κB induced by LMW-HA fragments and TLR-2,4 promotes fibrosis by orchestrating local inflammatory reactions and altering protease-antiprotease balance maintaining the fibrotic responses [87].

## Discussion

The pathogenesis of bleomycin-induced pneumonitis is associated with multiple mechanisms, including oxidative damage, protease-antiprotease imbalance [38], caveolin deficiency [78], TGF-β1 [69], and genetic susceptibility [88]. Initially, the ECM was considered to be a simple scaffold providing structural support to lung airways. However, recently, the ECM components have been observed to be a major determinant of cell behavior, fate, and function [37].

In the present study, we elaborate on the dynamic role of ECM and LMW-HA fragments in regulating the epithelial injury/repair processes. In the early phase, LMW-HA alerts the immune system of a breach in tissue integrity [23] and activates TLR-2,4, alveolar macrophages, and NF-κB signalling, resulting in inflammation. TLR-4 mRNA subsequently downregulates and shifts the TLR-2/TLR-4 balance to more than 1. This predisposes to the progression of inflammation to fibrosis [14, 80, 89] and results in a progressive increase in lung hydroxyproline levels [86]. Thus, the ECM-driven LMW-HA-TLR-2,4-NF-κB pathway defines the extent of cellular macrophage infiltration and parenchymal matrix remodelling. They are reflective of the state of tissue integrity and may serve as biomarker of active fibrosis in chronic lung diseases and as potential therapeutic targets.

In 2020, efforts have been made to understand the pathophysiology of the novel coronavirus patients who are predisposed to develop chronic lung disease following COVID-19. These patients have lung inflammation with activation of NF-kappa B (NF-κB) transcription factor, in lung macrophages [90], release of inflammatory cytokines (IL-1β,6, TNF-α), induction of HA synthase 2 in lung AEC, endothelium and fibroblasts, accumulation of prominent hyaluronan exudates in the alveolar spaces, and progression to acute respiratory distress syndrome, [7]. High molecular weight HA predominates in most tissues under healthy conditions, whereas fragmented low molecular weight HA polymers predominate at sites of active inflammation [91], thereby suggesting that adjuvant treatment targeting hyaluronan, such as intranasal administration of exogenous hyaluronidase or HA inhibitor (4-methylumbelliferone (4-MU) [60] may be a promising approach to reduce mortality in critically ill covid-19 patients [7]. Similarly, the immunomodulation of NF-κB activation and inhibitions of NF-κB (IκB) degradation may result in a reduction of the cytokine storm and have been suggested as a potential therapeutic target for severe COVID-19 [90].

The strong binding of the SARS-COV-2 spike protein with Toll-like receptors-1,4,6 and especially with TLR-4 causes an intense exacerbation of the host immune response with release of interleukin-6 (IL-6) and tumor necrosis factor-alpha (TNF-α), and enhanced severity of COVID-19 pathology [73]. The TLRs are pattern recognition receptors which recognize pathogen-associated molecular patterns (PAMPs) as well as endogenous DAMPs such as hyaluronan and trigger the innate immune response [92]. TLR-4 activation kills the microbes but can cause DAMP associated host tissue damage as has been previously reported [93, 94]. Tissue damage is initiated by the myeloid differentiating primary response gene 88 (MyD88)-dependent or the MyD88-independent pathways [95] leading to macrophage, natural killer cell, mast cell recruitment and their release of several interleukins, interferons, reactive oxygenspecies (ROS), and reactive nitrogen species (RNS) [96]. Moreover, the TLR4-NF-κB pathway is central towards promoting infection-induced lung injury in aging patients with comorbidities such as diabetes, atherosclerosis, obesity, and hypertension, thus suggesting the utility of therapeutic targeting of TLR-4 pathway by compounds such as statins, ACE inhibitors, opioids, and steroids in COVID-19 [73].

## Conclusions

The lung parenchymal hyaluronan fragments and TLR2/TLR4 balance form the critical link between AEC apoptosis [14], activation of innate immune response, and development of cytokine storm, inflammation, and lung fibrosis in both infectious and non-infectious lung injury. The LMW-HA-TLR-2,4-NF-κB pathway should be explored as a biomarker and for its therapeutic potential, in controlling the severity of lung inflammation and its progression to lung fibrosis.

## Data Availability

Not applicable

## Abbreviations

PF: Pulmonary fibrosis
SARS-CoV-2: Severe acute respiratory syndrome coronavirus 2
ECM: Extracellular matrix
LMW-HA: LMW-hyaluronan
DAMP: Damage-associated molecular patterns
TLR: Toll-like receptor
NF-κb: Nuclear factor-kappa B
AECs: Alveolar epithelial cells
EMT: Epithelial–mesenchymal transition
IL: Interleukins
TNF: Tumor necrosis factor
HAS2: HA synthase 2
DAD: Diffuse alveolar damage
ARDS: Acute respiratory distress syndrome
MMP: Matrix metalloproteinases
IRAK-1: IL-1R-associated kinase 1
TRAF: TNF receptor-associated factor
JNKs: Jun N-terminal kinases
MAPKs: Mitogen-activated protein kinases
TGF-β1: Transforming growth factor-β1
PDGF-1: Platelet-derived growth factor-1
MIP-1: Macrophage inflammatory protein-1
